# Guidance about age‐friendly outdoor exercise equipment and associated strategies to maximise usability for older people

**DOI:** 10.1002/hpja.367

**Published:** 2020-06-20

**Authors:** Pazit Levinger, Maya Panisset, Helen Parker, Frances Batchelor, Marian Tye, Keith D. Hill

**Affiliations:** ^1^ National Ageing Research Institute Melbourne Vic. Australia; ^2^ Institute for Health and Sport Victoria University Melbourne Vic. Australia; ^3^ Department of Health and Human Services Community Based Health Policy Health and Wellbeing Melbourne Vic. Australia; ^4^ School of Design and Built Environment Curtin University Perth WA Australia; ^5^ Department of Primary Care and Allied Health Rehabilitation, Ageing and Independent Living Centre Monash University Melbourne Vic. Australia; ^6^ School of Physiotherapy and Exercise Science Curtin University Perth WA Australia

**Keywords:** ageing, built environment, local government, older people, physical activity, exercise

## Abstract

Outdoor exercise equipment has become popular as important environmental infrastructure to provide opportunities for physical activity and social connectedness in public settings. With higher sedentary behaviour and physical inactivity reported among older people, infrastructure changes and safe environments that promote older peoples’ health and mobility are required. Due to ageing‐related functional decline and health conditions associated with ageing, older adults may have special physical needs that require careful consideration when choosing outdoor equipment. However, limited information is available regarding the suitability of the types of exercise equipment for older people. This commentary provides further information on the type of equipment available, its functionality and suitability for older age populations and key considerations for the decision‐maker involved in selecting, installing and supporting community use of outdoor exercise equipment. Recommendations on what is required to maximise usability from a system or organisational‐based approach using research evidence is also discussed. Older people are more susceptible to the negative influences of their local environment and outdoor neighbourhood conditions. Consequently, the age‐friendliness and suitability of the outdoor exercise equipment characteristics, location and settings may facilitate older adults’ engagement in physical and social activities.

## 1 INTRODUCTION

Adequate physical activity is important for public health and disease prevention, and should be promoted globally.^1^ Physical inactivity has been identified as the fourth leading risk factor for global mortality.^1^ Levels of physical activity tend to decline in older age, with only 25% of people aged 65 and over undertaking guideline recommendations for physical activity.^2^ Increasing physical activity demands a system‐based approach as identified by the World Health Organisation (Global Network for Age‐friendly Cities and Communities), where one of the key objectives of the action plan is to create active environments.^3^ Active environments encompass the creation and maintenance of environments in which people of all ages have access to safe places and spaces, in their cities and communities, in which to engage in regular physical activity, according to ability.^3^ Strengthening the policy, regulatory and design guidelines and frameworks to promote sports and recreation in outdoor spaces is important to enable residents and visitors with diverse abilities to be physically active.

The world population is ageing. In Australia, the number of older people is projected to increase from approximately 3.8 million in 2017 to 8.8 million in 2057 (22% of the population).^4^ Given the projected increase in the number of older people in coming years, there will be increased demand on state and local governments to strategically focus on positive ageing and inclusiveness through the creation of age‐friendly environments and health promotion interventions. Being active outdoors is beneficial for mental, physical and social health across the lifespan.^5^, ^6^ In older age in particular, therapeutic landscape (nature and greenery) can positively impact on physical, mental and social health, in which experiences of nature provoke feelings of renewal, restoration and spiritual connectedness.^7^ With higher sedentary behaviour and physical inactivity reported among older people, local governments are well positioned to make infrastructure changes that promote older peoples’ health and mobility by creating outdoor spaces for older people to engage in physical activity.

Outdoor exercise equipment (often referred to as outdoor fitness equipment, outdoor gym, fitness zone, seniors playground and seniors exercise park) has become popular as an important environmental infrastructure to provide opportunities for physical activity and social connectedness in public settings at no cost.^8^, ^9^, ^10^ The concept of promoting healthy living behaviour through the installation of outdoor exercise equipment has demonstrated some promise in the facilitation and improvement of physical activity patterns and also as a cost‐effective investment for increased park utilisation and park‐based physical activity.^8^, ^11^ Due to ageing‐related functional decline and health conditions associated with ageing, older adults may have special physical needs that require careful consideration when choosing outdoor equipment. Older people are more susceptible to the negative influences of their local environment and outdoor neighbourhood conditions. Consequently, the age‐friendliness and suitability of outdoor exercise equipment characteristics, its location and settings are important to facilitate older adults’ engagement in physical and social activities.

With the increasing popularity of outdoor exercise equipment, there are a wide range of exercise equipment options available, yet little information on how to choose the right equipment to suit the targeted primary end‐user group.^12^ Specifically, limited information is available regarding the suitability of these types of exercise equipment for older people. Whilst outdoor exercise equipment generally targets adults, their nonspecific nature means that their suitability for older people might be questionable. This commentary aims to provide further information on the types of equipment available, its functionality and suitability for older age populations, and key considerations for the decision‐maker involved in selecting, installing and supporting community use of this equipment. This commentary also provides recommendations on what is required to maximise usability from a system or organisational‐based approach using research evidence.

## 2 WHAT TO CONSIDER WHEN CHOOSING OUTDOOR EXERCISE EQUIPMENT FOR OLDER PEOPLE

There are several factors that need to be considered for suitable equipment selection and purchase. These include: the type of equipment, its functionality and limitations, the targeted demographic, the primary end‐users’ physical needs and the physical built environment. Other factors to increase participation and usage are also discussed. A summary of recommended considerations check list is provided in Table 1.

**TABLE 1:** Recommended check list

Physical activity types to be targeted by the outdoor exercise equipment	
	✓Day to day movements ✓Functional strength ✓Joint movement and flexibility ✓Balance ✓Aerobic fitness
Built environment – location	
	✓Close proximity to residential area, amenities and community hubs ✓Proximity to other sport and recreational opportunities ✓Easily accessible by foot and other mode of transports ✓Co‐location near children's playgrounds
Settings and safe ground surface	
	✓Benches and sheltered resting areas ✓Water fountain ✓Nonslip and compliant surface (Softfall or equivalent), ✓Safe sidewalk/trials ✓Shade cover
Increase participation and engagement	
	✓Clear instructions and signage ✓On‐site labelling/graphics ✓Organised programs/activities ✓Designated times for older people classes ✓Supervision and instructional sessions ✓Effective communication, marketing and information ✓Age‐friendly senior ambassadors/champions

### 2.1 Types of common outdoor equipment, targeted users and safety considerations

**FIGURE 1:**
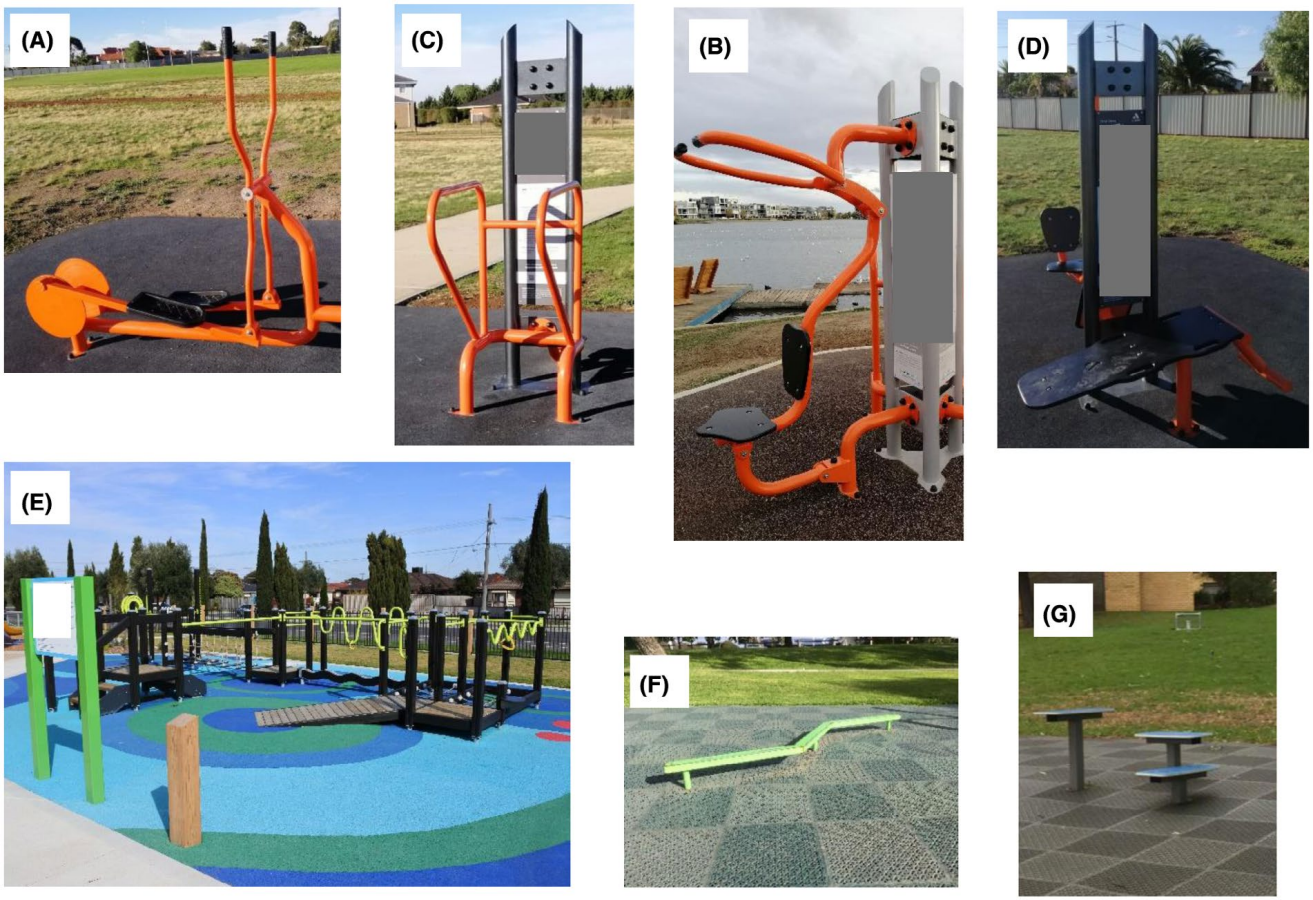
Type of common outdoor exercise equipment: aerobic/cardio machine (A – cross trainer); dynamic resistance gym based machine (B – pull down); static gym based equipment (C – pull/push ups; D – sit ups bench); multiple stations of unstable surfaces, balance beams, mobility (E – Seniors Exercise Park); walking beam (F); jump boxes (G)

A summary of the type of equipment, targeted users and associated safety considerations is provided in Table 2. Outdoor equipment can be classified as follows:

- Dynamic aerobic/cardio machine ‐ includes circular/elliptical motion, aiming to challenge the cardiorespiratory system. Examples of cardio outdoor equipment include the skywalk, bicycle, ski walker, cross trainer (Figure 1A).
- Dynamic resistance gym‐based machine ‐ mimics the conventional indoor gym equipment where it targets strengthening of the skeletal muscles. This equipment includes pushing or pulling against resistance (body weight or equipment weight as set by the manufacturer). Examples include leg press, pull/push up, shoulder/chest press (Figure 1B).
- Other static gym‐based equipment ‐ includes pull or push up bars and benches (eg, sit up) that require the user to work against his/her body weight (Figure 1C,D)
- Mobility and stretching equipment includes equipment stations that target flexibility and range of motion and requires movement/sliding of body parts – (shoulder/arm arch, core twister)
- Seniors Exercise Park – includes multiple equipment stations (multimodal exercise equipment) that target mobility, functional movement, unstable and uneven balance surfaces (Figure 1E).
- Other type of equipment may include walking beams (Figure 1F) and elevated jump box/bars that target agility (Figure 1G).

**TABLE 2:** Type of equipment, targeted users and associated safety considerations

Type	Functionality	Examples	Targeted primary user	Safety considerations	Limitations	Other considerations for older people
Aerobic machine	Cardiorespiratory system	Sky walker, Cross/elliptical trainer, stationary bike	Adults		No resistance, no adjustable pieces. No progression of exercise difficulty is possible	
Dynamic resistance gym‐based machine	Strength	Leg press, chest press, pull down	Adults Experienced/fit older adults	Users need to be able to lift body weight pull/push against machine weight	Fixed movement based on manufacture. No adjustable points/elements for different individual needs/body size/dimension No progression of exercise difficulty is possible	
Static gym‐based machine		Pull/push up bars, benches	Adults Experienced/fit older adults	Users need to be able to lift body weight. Users need to be able to do transfer movements unaided (eg, standing up from a lying position)		
Balance beams, agility equipment			Adults	Not safe for older people if no handrail provided	Agility/jump boxes are high boxes > 30 cm, which limit usability for those who are unable to jump safely to/from a high platform	
Senior Exercise Park	Multiple stations of range of motion, functional strength, balance, cardiorespiratory fitness	Shoulder arch, core twister, sit to stand, balance beam, unstable walkway bridge, finger steps, stairs, step up platform.	Adults and older people	Hand rails/bar are provided for each equipment piece	Can be used by children; a priority use by older people should be indicated	Multigenerational (can be used by children and adults alike) Exercises can be adjusted/modified for various physical capabilities
Others ‐ stand‐alone static equipment	Flexibility and strength	Stretching station or step up platforms	Adults and older people	Handrail/hand support bar is needed for safety	Step height higher than the accepted standard height (22.5 cm) will limit usability	

The above items of exercise equipment have some limitations. Most equipment comes with manufacturer restrictions in terms of movement, minimum age restrictions, weight and shape/size. Adjustments for individual ability or body dimensions (height, body limbs length) are often not possible. For example, the dynamic gym‐based machines do not allow adjustment of parts (eg, seat), they also require users to have the strength to push/pull against the weight of the equipment. Moreover, adjustment to various resistance levels might be limited, hence, individualised exercise regression or progression (decrease or increase the demand of an exercise or movement through minor changes such as increase/decrease weight/resistance) cannot be made. If users do not possess the level of strength required to move the equipment, they will be unable to use it or they will use it incorrectly (poor technique). Similarly, the aerobic machines often require minimum effort for operation with no ability to change. Consequently, the frequent lack of adjustable resistance or progressively adaptable elements do not allow for appropriate and sufficient muscle stimulus to suit different individuals.^13^ Outdoor equipment that incorporates stations/equipment pieces that mimic day‐to‐day movements and activities (eg, sit to stand, stair climb, reaching) might be of greater benefit for older people due to its emphasis on functional strength and joint range of movement required for daily functioning and independence.^14^ Individualising exercises to suit various abilities for older people might be possible using such equipment. An example of individualisation of an exercise program with various levels of progression to suit older people can be found using the Seniors Exercise Park equipment.^15^, ^16^ However, it is important to note that for older people who lack the knowledge and or the experience in how to exercise, providing a professional exercise session/program with an exercise instructor might be required, at least in early stages of acclimatisation for safe and correct use of the exercise equipment. This is also important as inappropriate usage behaviour (operation of outdoor exercise equipment not according to manufacture instructions) might pose potential safety risks.^17^

From a safety perspective, another important aspect to consider with outdoor exercise equipment options that aim to improve balance performance (eg, balance platform/beam, unstable surfaces) is the inclusion of safety rails (as provided in some equipment kits such as the Seniors Exercise Park equipment) and the potential need for professional advice. While using this type of equipment is likely to be safe for healthy older people to exercise on, older people with balance or mobility impairments may require health professional advice and supervision. Appropriate signage, safety disclaimer messages and promotion of this information (through avenues such as local council information) disseminated to areas around where these type of parks are located are required to ensure that use is limited to those who can safely use the park equipment.

### 2.2 The recommended type and duration of physical activity for older people

The recommended physical activity guidelines for older people advise older people to engage in at least 30 minutes of physical activity daily that incorporates moderate fitness activities (for cardiorespiratory health), strength activities (for muscle and bone health), flexibility activities (to maintain joint range of motion) and balance activities (to reduce falls).^18^, ^19^ The type and number of outdoor equipment items varies among sites and locations and the rationale for the equipment selection is often unclear. Given the recommended type of physical activity for older people, the selection and guiding principle should take into account these physical activity recommendations, with the inclusion of equipment for aerobic fitness, upper and lower limb strength, balance and flexibility. However, most common outdoor equipment includes dynamic aerobic cardio machines and dynamic resistance gym‐based equipment, with few equipment items that specifically target balance and flexibility.^10^, ^20^ Improving balance is one of the key recommendations for falls prevention in older people, where balance exercises can reduce the rate of falls by up to 39%.^21^ Outdoor equipment that provides a safe but challenging environment for balance training (eg, the Seniors Exercise Park equipment^20^) is, therefore, important to consider in the design of active outdoor space for older people.

Recent studies reported physical benefits for older people using outdoor exercise equipment. Six weeks of combined aerobic and resistance training using dynamic aerobic and resistance gym‐based machines resulted in improved upper and lower muscular strength, endurance and physical function.^22^ In another study, an 18‐week exercise program using the Seniors Exercise Park resulted in improved balance, strength and physical function for older people with high risk of falls. The Seniors Exercise Park program was also associated with social benefits for older people in the community.^20^, ^23^ Both exercise interventions, using different type of equipment, were structured and supervised by qualified staff with incremental progression of the exercises allowing optimal improvement and physical benefits for older people. This provides promising results for the use of outdoor exercise equipment for older people to improve their health.

### 2.3 The physical built environment – location and settings

The location and settings of the exercise equipment is important to maximise usage. Often exercise equipment is installed along walking tracks, public parkland and local community public open spaces. The choice of location can have a significant impact on usage. In order to increase physical activity and park use by older adults, the equipment is best installed in areas that are proximal to a large target population and that are easily accessible^8^ (eg, location at a community park near a seniors centre). Other important aspects to consider are the physical nature of the environment and how it can impact on who can access it. For example, installation of outdoor equipment along tracks will require users to have the physical ability to access it by foot, therefore, older people who are unable to walk unaided or for long distances are unlikely to be able to access, and therefore use, the equipment. To increase older peoples’ participation, easy access, close by amenities and community hubs (community centre, senior groups) are important to consider.^24^

### 2.4 Surrounding surface and pedestrian infrastructure

The ground surfaces surrounding the exercise equipment should be safe in all weather conditions. The built environment can contribute to falls where most outdoor falls (73%) are precipitated by environmental factors, such as tripping on uneven surfaces or slipping on objects, which have been reported to usually occur on sidewalks, curbs and in the street.^25^ Maintaining safe pedestrian infrastructure (sidewalk, trails) to and from the outdoor equipment can, therefore, minimise falls risk.

Nonslippery rubber (softfall or equivalent) is often used under and around the exercise equipment as it is designed to absorb impact from falls and protect against injury in a playground environment. It consists of a dual layer structure, the wear layer which is the visible top surface, and the shock absorbing layer underneath, which is typically made from recycled rubber. This can reduce the risk of injury if falls occur as well as support the environment (usage of recycling strategies to mitigate against climate change).

### 2.5 Weather elements

Older people in particular are vulnerable to extreme weather.^26^, ^27^ They are more sensitive to changes in the environment, which is a by‐product of a lower physiological reserve capacity, slower metabolism, and a more slowly responding immune system.^26^ This makes their body less able to tolerate stress such as posed by the environment (extreme hot/cold weather). The climate conditions can vary between locations and can play a major role in safety (ie, wet/hot weather). A shade cover (preferably waterproof cover) over the exercise equipment and a drinking fountain are recommended for participant's health and safety. Avoiding peak temperature and ultraviolet light exposure should also be practiced for organised, structured classes.^28^ The presence of benches and sheltered resting areas are also important to allow the opportunity to rest, especially for those with reduced functional capacities.^29^

### 2.6 Intergenerational outdoor space

Older people often provide care for their grandchildren. Approximately, 18% of children under 13 years are cared by their grandparents.^30^ This presents an opportunity for a shared experience, either using the same equipment, or using co‐located outdoor equipment. Designing an outdoor space that includes both outdoor equipment for older people and playground for children is an important consideration to allow all generations to be physically active. Co‐locating exercise parks targeting older people near children's playgrounds can further foster intergenerational interaction. However, consideration of safety of all age groups is important. Public space can be accessed and used by all residents. While there are no local laws relating to priority use of public facilities by different groups, often local governments’ position is that the group for which the equipment is designed has priority use. This is important, as access by children at times when older people are using equipment may pose a safety risk. During designated times where structured, organised activities are taking place, precedence should be given to these groups. Communication and information for public awareness should be provided by local governments. Creative communication using various platforms that encourage and support sharing outdoor spaces for all ages is recommended for community cohesion.

### 2.7 How to facilitate engagement of older people

There are several ways to increase and facilitate community and user engagement. First, the inclusion of signage is important in making the park salient, with effective cues and instructions to increase physical activity.^31^, ^32^ Inadequate instructional support and lack of relevant information for users can be barriers to using outdoor exercise equipment.^10^ Providing clear instructions, on‐site labelling and graphic illustrations can assist users to understand what to do and be independent users as well as assist in increasing efficient use of the equipment to achieve positive health outcomes.^33^, ^34^ For older people, providing supervision and instructional sessions by qualified exercise instructors can also positively engage older adults in outdoor exercise equipment use.^23^, ^35^ A recent study from Asia reported independent users spent less than 9 minutes on all outdoor exercise equipment combined, highlighting the lack of sufficient time spent to meet the minimum duration for physical activity recommendations.^36^ Consequently, incorporating education and information around correct equipment use, adequate amount and intensity of physical activity are also required to achieve health benefits.

### 2.8 How to increase participation/uptake and adherence

Social connectedness has been identified as one of the key themes of users to attend and use outdoor exercise equipment.^10^ Investment in park/site programming such as organised events or activities seems to be associated with higher park usage.^37^ In fact, overall park use and physical activity was reported to decline by 39% due to a reduced number of organised programs in Southern California.^38^ For older people, the social element of engaging in activities, fun and enjoyment are key motivators for older people to take part in physical activity^39^ and they prefer to exercise with their same age group.^40^ Training age‐friendly senior ambassadors/champions to act in community leadership roles and as role models can also greatly improve communication and engagement of older people to increase participation in programs, activities and events. Similar strategies have been employed by the National Heart Foundation Walking program which resulted in sustained engagement in outdoor physical activity (80% were still active after 6 months).^41^

Designing outdoor activities with the use of outdoor exercise equipment should facilitate the opportunities for social interaction, the creation of new relationships and provide social support to motivate and encourage older people to engage in physical activities.^23^, ^42^ If the equipment is located close to a community centre, prior agreements can be made for the participants to use the space to share food and drink following the session. Similarly, scheduling regular times to meet and exercise (eg, organised session), so that people know when others will be there, can also facilitate ongoing participation.

Other important aspects to increase uptake of outdoor physical activity and community engagement include targeted marketing and promotion.^31^, ^33^ Consequently, to maximise outreach for older people to benefit from outdoor exercise equipment, a multilayer approach should be considered that incorporates targeted marketing, information, promotion and organised group activities.^43^ Local governments plan and deliver services in health, recreation, planning and building control, and human and community services. As such, local governments, through their varying roles and levels of community engagement, can play a significant role in designing, facilitating and delivering safe outdoor active space for older people.

## 3 CONCLUSION

Commonly, outdoor exercise equipment is often suitable for able‐bodied adults with little consideration for the physical needs of older people. The design of age‐friendly outdoor spaces should be incorporated into decision‐making using a set of age‐friendly considerations, where the chosen equipment is safe, targets balance, functional strength and day‐to‐day movements and activities. Providing clear instructions and information, as well as promotion, organised activities and programs are important factors in maximising engagement and social connectedness of older people in the community in order to achieve positive health outcomes.

## 4 CONFLICT OF INTEREST STATEMENT

The authors declare no conflicts of interest.
